# Ganoderic acid A attenuates lipopolysaccharide-induced lung injury in mice

**DOI:** 10.1042/BSR20190301

**Published:** 2019-05-24

**Authors:** Bing Wan, Yan Li, Shuangshuang Sun, Yang Yang, Yanling LV, Li Wang, Meijuan Song, Meizi Chen, Chengjiang Wu, Hangcheng Pan, Xiuwei Zhang

**Affiliations:** 1 Department of Respiratory and Critical Care Medicine, the Affiliated Jiangning Hospital of Nanjing Medical University, Nanjing 210002, China; 2 Jiangsu Province Key Laboratory of Geriatrics, Department of Geriatric Medicine, the First Affiliated Hospital of Nanjing Medical University, Nanjing 210029, China; 3 Department of Respiratory Medicine, Jinling Clinical Medical College of Nanjing Medical University, Nanjing 21002, China; 4 Department of Comprehensive Internal Medicine, Chenzhou First People’s Hospital, Chenzhou 423000, China; 5 Department of Immunology, Key Laboratory for Laboratory Medicine of Jiangsu Province, Jiangsu University Medical School, Zhenjiang 212013, China

**Keywords:** ganoderic acid A, lipopolysaccharide, lung injury, nuclear factor kappaB, rho-associated protein kinases

## Abstract

The present study aimed to investigate the protective effects of ganoderic acid A (GAA) on lipopolysaccharide (LPS)-induced acute lung injury. In mouse model of LPS-induced acute lung injury, we found that GAA led to significantly lower lung wet-to-dry weight ratio and lung myeloperoxidase activity, and attenuated pathological damages. In addition, GAA increased superoxide dismutase activity, but decreased malondialdehyde content and proinflammatory cytokines levels in the bronchoalveolar lavage fluid. Mechanistically, GAA reduced the activation of Rho/ROCK/NF-κB pathway to inhibit LPS-induced inflammation. In conclusion, our study suggests that GAA attenuates acute lung injury in mouse model via the inhibition of Rho/ROCK/NF-κB pathway.

## Introduction

Acute lung injury (ALI) has a high mortality of up to 40% and is a serious respiratory disease characterized by neutrophils infiltration, pulmonary edema, and inflammation [1]. While the pathogenesis of ALI remains unclear, it is accepted that during ALI the injury of the alveolar epithelium and capillary endothelium would cause airspace edema and inflammation due to increased alveolar barrier permeability [1]. Therefore, it is urgent to explore effective treatment for ALI by targeting inflammation.

*Ganoderma lucidum* has been widely used in traditional Chinese medicine for a variety of diseases such as hypertension, hepatitis, and immunological disorders. Ganoderic acid A (GAA) is one of triterpenoid extracts of *G. lucidum* with a range of biological activities [2,3]. In particular, a recent study reported that GAA has anti-inflammation activity to inhibit lipopolysaccharide (LPS)-induced secretion of inflammatory cytokine [4]. However, the efficacy of GAA to treat LPS-induced lung injury remains unclear.

Several studies have shown that ROCK pathway could regulate NF-κB activity to promote inflammatory response, while ROCK inhibitor inhibited the activation of NF-κB and the production of inflammatory cytokines [5–7]. Therefore, Rho/ROCK/NF-κB pathway is crucially involved in inflammation. In the present study, we examined the protective effects of GAA on LPS-induced acute lung injury and explored whether these effects are related to the inhibition of Rho/ROCK/NF-κB pathway.

## Materials and methods

### Reagents

GAA was provided by State Center for Standard Substances (Beijing, China) and the purity was >96%. Dexamethasone (Dex) and LPS were provided by Sigma-Aldrich, (St. Louis, MO, U.S.A.).

### Animals

Animal experiments followed the Guideline for the Care and Use of Laboratory Animals. Male BALB/c mice (weight 18–22 g) were purchased from Qinglongshan Animal Cultivation Farm (Nanjing, China) and maintained in standard conditions with 12-h light/dark cycle. The mice were divided randomly into five groups (*n*=10): (1) control group (phosphate-buffered saline, PBS), (2) LPS group, (3) LPS + Dex group (LPS + Dex, 2 mg/kg), (4) LPS + GAA group (GAA, 10 mg/kg), (5) LPS + GAA group (GAA, 20 mg/kg). The animals received PBS or drug intraperitoneally 1 h before intratracheal instillation of LPS at 20 μg (0.4 μg/μl). The animals were killed after 6 h.

### Collection of bronchoalveolar lavage fluid

The lungs were lavaged three times with 500 μl of PBS, and bronchoalveolar lavage fluid (BALF) was centrifuged at 300 ***g*** at 4°C for 10 min, and the supernatants were collected and stored at −80°C.

### Measurement of wet-to-dry ratio of the lungs

The trachea and esophagus were dissected from the right lungs, and the wet weight was determined. The lungs were incubated at 60°C for 48 h to determine dry weight, and the lung wet-to dry (W/D) ratio was calculated.

### Histopathology

The lungs were fixed in 4% formalin overnight, embedded in paraffin and cut into sections. Hematoxylin–Eosin (H&E) staining was performed as described previously [8]. Under a light microscope, inflammatory cells were counted to estimate leukocyte infiltration using the scoring system as: 0: no cells, 1: a few cells, 2: a ring of cells with 1 cell layer deep, 3: a ring of cells with 2–4 cell layers deep; and 4: a ring of cells with more than 4 cell layers deep.

### Measurements of myeloperoxidase and superoxide dismutase activity and malondialdehyde level

Myeloperoxidase (MPO) activity in the lung and superoxide dismutase (SOD) activity and malondialdehyde (MDA) level in BALF of the mice were measured using kits from Jiancheng Bioengineering Institute (Nanjing, China), following the manufacturer’s instruction.

### Measurements of cytokine levels

The levels of tumor necrosis factor-α (TNF-α), interleukin-1β (IL-1β), and interleukin-6 (IL-6) in BALF of the mice were determined using ELISA kits from Key GEN Biotech (Nanjing, China) following the manufacturer’s instruction.

### RhoA activity assay

About 25 μg of lung tissue was homogenized on ice and the homogenate was collected. RhoA activity in the homogenates was evaluated by Rho-GTP pulldown assay using Rho Activation Assay Biochem Kit (Cytoskeleton, Denver, CO, U.S.A.) following the manufacturer’s instruction.

### Western blot analysis

The lung tissues were lysed on ice in radioimmunoprecipitation assay buffer supplemented with 0.1% phenylmethylsulfonyl fluoride. The lysates were centrifuged at 12,000 rpm at 4°C for 10 min, and protein concentration was measured using BCA protein assay kit. The proteins were separated by polyacrylamide gel electrophoresis and transferred onto the membranes. After incubation with primary antibodies for Rho (#2177), ROCK-I (#4035), ROCK-II (#9029), p-IκBα (#2859), IκBα (4812), NF-κB p65 (#8242), and p-NF-κB p65 (#4764) (Cell Signaling, Danvers, MA, U.S.A.) at 4°C overnight, the membranes were incubated with secondary antibodies at room temperature for 2 h. The membranes were washed and developed using ECL kit (Pierce, Rockford, IL, U.S.A.). The band density was estimated using Image.plus5.1 program.

### Statistical analysis

All data were presented as mean ± standard error of the mean. Differences among groups were analyzed by one-way ANOVA followed by Tukey test. *P*<0.05 indicated significant.

## Results

### GAA decreased W/D weight ratio in the lungs

**Figure 1:**
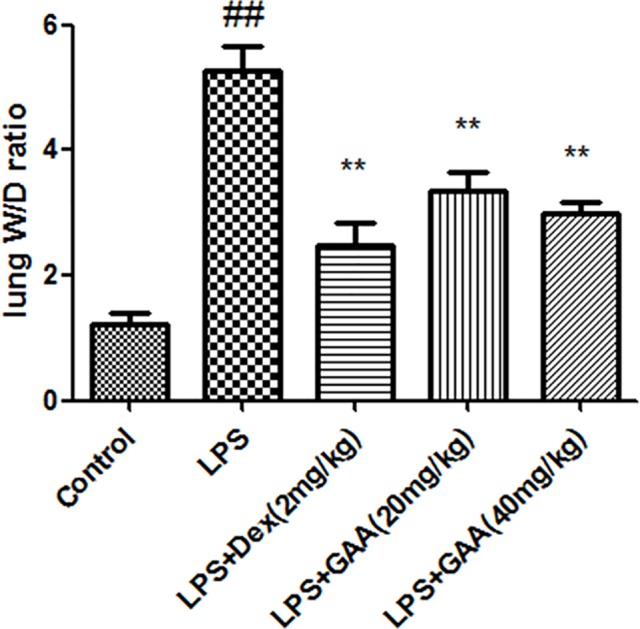
Effect of GAA on wet-to-dry weight ratio in lung tissues The lung tissues were dissected from each group of mice, and wet-to-dry weight ratio of the lungs was calculated. Values are expressed as means ± SEM (*n*=8). Compared with control: ^##^*P*<0.01; Compared with LPS:, **P<0.01.

First, we determined the effect of GAA on pulmonary edema. W/D weight ratio of the lungs was significantly higher in LPS model than in control group. However, GAA treatment led to lower W/D weight ratio in model group (Figure 1).

### GAA attenuated pathological changes in the lungs

**Figure 2:**
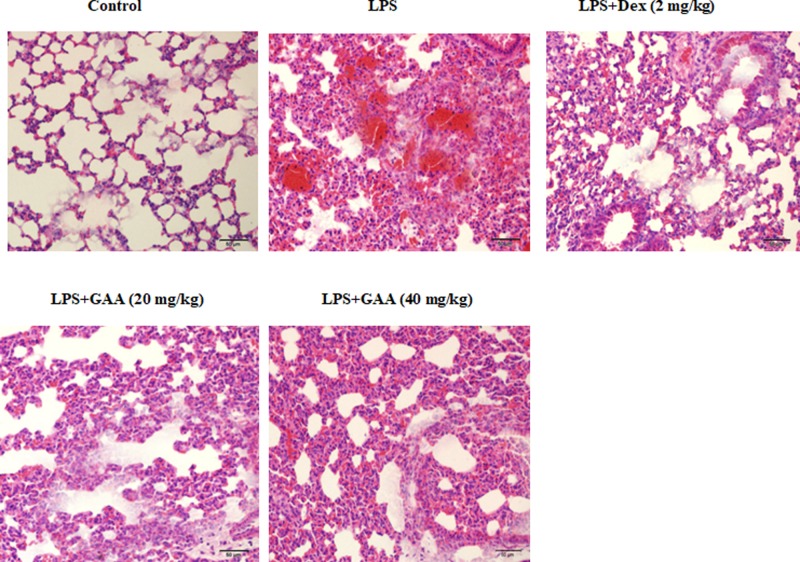
Effect of GAA on pathological changes in lung tissues Shown were representative images from at least three mice in each group. Compared with control group, significant neutrophil sequestration or infiltration in the lungs was observed in LPS group. In GAA- and Dex-treated groups, much less neutrophil sequestration or infiltration was observed. H&E staining (×200).

Next, we examined the effect of GAA on pathological damages in the lungs. H&E staining showed significant neutrophil sequestration or infiltration in the lungs of model group compared with the control group, but much less neutrophil sequestration or infiltration in the lungs of GAA group compared with model group (Figure 2).

### GAA decreased MPO activity and MDA level while increased SOD activity in the lungs and BALF

**Figure 3:**
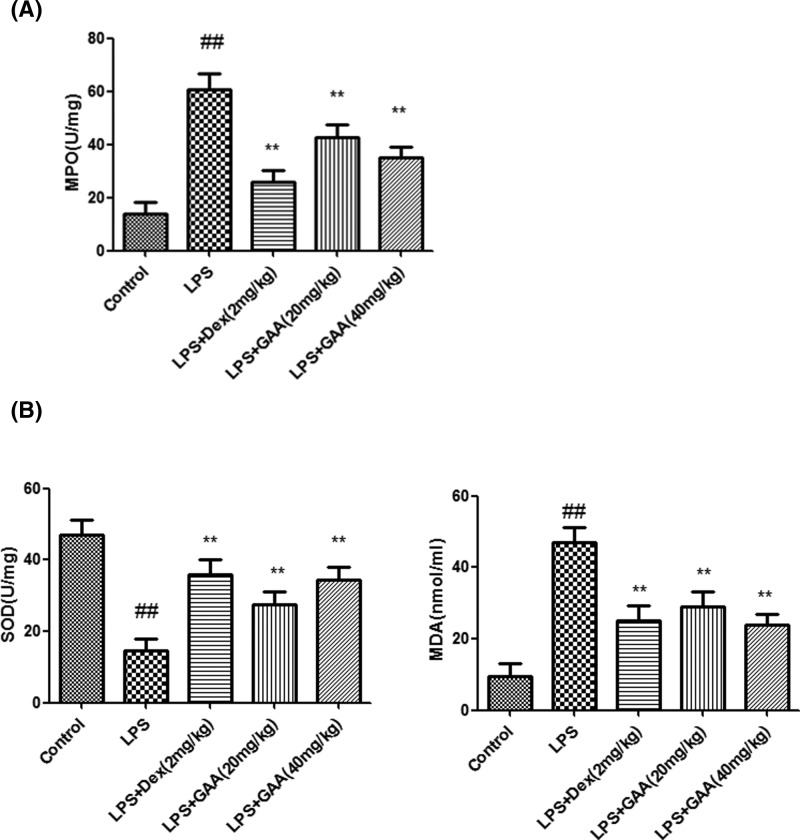
Effect of GAA on MPO activity in lung tissue (A), MDA level and SOD activity in BALF (B) The lung tissues or BALF were collected from each group of mice, and MPO activity, MDA level and SOD activity were measured. Values are expressed as means ± SEM (*n*=6). Compared with control: ^##^*P*<0.01; Compared with LPS: **P<0.01.

Compared with control group, MPO activity in the lung and MDA level in BALF significantly increased in model group. However, GAA remarkably decreased MPO activity in the lung and MDA level in BALF of model group. However, SOD activity in BALF was significantly lower in model group than in control group, while GAA remarkably increased SOD activity in BALF of model group (Figure 3).

### GAA decreased TNF-α, IL-6, and IL-1β levels in BALF

**Figure 4:**
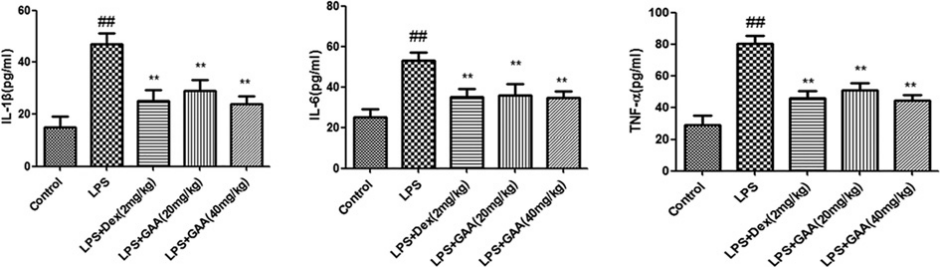
Effect of GAA on TNF-α, IL-6, and IL-1β levels in BALF The BALF was collected from each group of mice, and TNF-α, IL-6, and IL-1β levels were measured by ELISA. Values are expressed as means ± SEM (*n*=6). Compared with control: ^##^*P*<0.01; Compared with LPS: **P<0.01.

Next, we detected TNF-α, IL-1β, and IL-6 levels in BALF of each group. While TNF-α, IL-1β, and IL-6 levels increased significantly in BALF of the model group compared with control group, GAA remarkably decreased their levels in BALF of model group (Figure 4).

### GAA inhibited Rho/ROCK/NF-κB signaling pathway in the lungs

**Figure 5:**
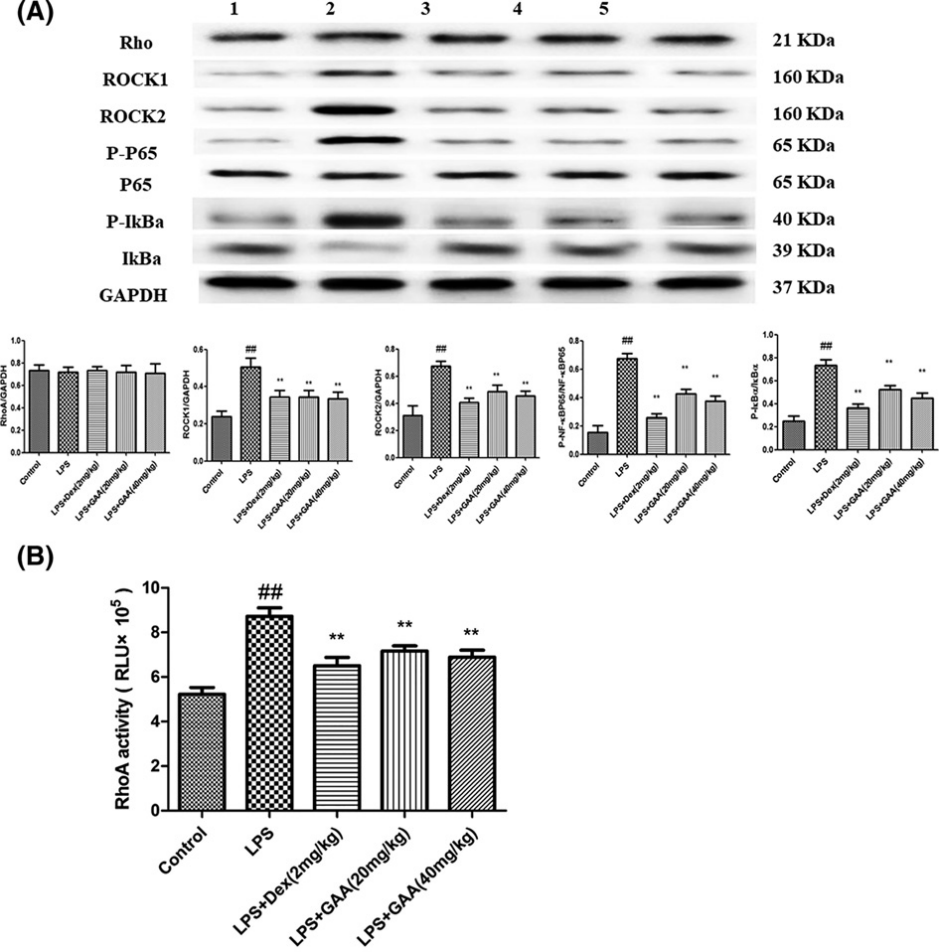
Effect of GAA on Rho/ROCK/NF-κB signaling pathway in lung tissues (**A**) The lung tissues were dissected from each group of mice, and protein levels were detected by Western blot analysis. (1) control group (PBS treated), (2) LPS group, (3) LPS + dexamethasone group (LPS + Dex, 2 mg/kg), (4) LPS + 20 mg/kg GAA group, (5) LPS + 40 mg/kg GAA group. (**B**) RhoA activity in the lung tissues of each group. Values are expressed as means ± SEM (*n*=6). Compared with control: ^##^*P*<0.01; Compared with LPS: **P<0.01.

To explore the mechanism by which GAA attenuated acute lung injury, we focused on Rho/ROCK/NF-κB pathway because it is crucially involved in inflammation. We found that the levels of phosphorylated NF-κB, IκBα, ROCK-I, and ROCK-II increased significantly in lung tissues of the model group, but Rho expression level did not change compared withy control group. However, GAA reduced the levels of these proteins in lung tissues of the model group (Figure 5A). Furthermore, RhoA activity assay showed that RhoA activity of lung tissue in model group significantly increased compared with control group, but GAA significantly inhibited RhoA activity in model group (Figure 5B).

## Discussion

*Ganoderma lucidum* has been widely used in traditional Chinese medicine for the prevention and treatment of a variety of human ailments, including hypertension, chronic hepatitis, and immunological disorders [9]. GAA is one of triterpenoid extracts of *G. lucidum* with many pharmacological activities. Glucocorticoids are used extensively in the treatment of infectious disorders, and Dex is one kind of glucocorticoids which is used widely in the treatment of pulmonary infection. In the present study, we evaluated the effects of GAA acute lung injury in mouse model by using Dex as a positive control.

Lung volume reduction, lung ventilation/blood imbalance, and even progressive hypoxia and respiratory distress are observed during ALI. LPS causes similar pathological process of ALI and is widely used to induce ALI [10]. In the present study, we employed LPS-induced ALI model to show that GAA and Dex significantly decreased lung W/D weight ratio, MPO activity, MDA content and the levels of TNF-α, IL-1β and IL-6, while increased SOD activity in LPS-stimulated mice. Moreover, pathological analysis showed that GAA attenuated acute lung injury.

MPO represents the activation of polymorphonuclear neutrophil and induces oxidative stress and inflammatory response [11,12]. We found that MPO activity in lung tissues increased significantly after stimulation by LPS. However, in GAA group, the MPO activity in the lung was decreased. Many studies have shown that lipid peroxidation reaction will produce MDA, which causes cross-linking and polymerization of proteins or nucleic acids and leads to cytotoxicity [13]. Our data showed that GAA decreased the content of MDA. SOD is an important antioxidant that can eliminate free radical. Our results indicated that GAA increased lung SOD activity in model group. As the positive control, dexamethasone increased SOD activity significantly, consistent with previous report [14]. During acute lung injury several inflammatory cytokines are produced, such as TNF-α, IL-1β, and IL-6. In the present study, LPS induced the production of TNF-α, IL-6, and IL-1β in BALF, but their production was inhibited by GAA.

Finally, we explored the mechanism by which GAA provided protection against LPS-induced acute lung injury. Numerous external stimuli including LPS activate NF-κB signaling pathway, these stimuli promote the phosphorylation and degradation of IκBα, thus releasing the active NF-κB into the nuclei to drive gene expression [15,16]. ROCK isomers ROCK I and ROCK II are important downstream molecules of Rho [17]. ROCK is crucially involved in inflammation [18]. We found that LPS increased the activity of RhoA, the activation of NF-κB, and the levels of ROCK-I and ROCK-II in the lung tissues. However, LPS-induced up-regulation of these proteins could be abrogated by GAA. In conclusion, our results suggest that GAA could exert protective effects in mouse model of LPS-induced acute lung injury through inhibiting Rho/ROCK/NF-κB pathway. However, further studies in ALI patients are needed to evaluate the efficacy of GAA.

## Abbreviations

ALI: acute lung injury
BALF: bronchoalveolar lavage fluid
Dex: Dexamethasone
GAA: ganoderic acid A
H&E: Hematoxylin–Eosin
IL-1β: interleukin-1β
IL-6: interleukin-6
LPS: lipopolysaccharide
MDA: malondialdehyde
MPO: myeloperoxidase
SOD: superoxide dismutase
TNF-α: tumor necrosis factor-α
